# Real-Time Induction of Macrophage Apoptosis, Pyroptosis, and Necroptosis by *Enterococcus faecalis* OG1RF and Two Root Canal Isolated Strains

**DOI:** 10.3389/fcimb.2021.720147

**Published:** 2021-08-26

**Authors:** Danlu Chi, Xinwei Lin, Qingzhen Meng, Jiali Tan, Qimei Gong, Zhongchun Tong

**Affiliations:** ^1^Hospital of Stomatology, Guanghua School of Stomatology, Sun Yat-sen University, Guangzhou, China; ^2^Guangdong Provincial Key Laboratory of Stomatology, Sun Yat-sen University, Guangzhou, China

**Keywords:** apoptosis, pyroptosis, necroptosis, PANoptosis, *Enterococcus faecalis*, macrophage

## Abstract

To investigate the effects of two *Enterococcus faecalis* root canal isolated strains (CA1 and CA2) and of the OG1RF strain on apoptosis, pyroptosis, and necroptosis in macrophages. The virulence factors of *E. faecalis* CA1 and CA2 pathogenic strains were annotated in the Virulence Factors Database (VFDB). *E. faecalis* CA1, CA2, and OG1RF strains were used to infect RAW264.7 macrophages (MOI, 100:1). We assessed the viability of intracellular and extracellular bacteria and of macrophages at 2, 6, and 12 h post-infection. We used a live cell imaging analysis system to obtain a dynamic curve of cell death after infection by each of the three *E. faecalis* strains. At 6 and 12 h post-infection, we quantified the mRNA expression levels of PANoptosis-related genes and proteins by RT-qPCR and western blot, respectively. We identified ultrastructural changes in RAW264.7 cells infected with *E. faecalis* OG1RF using transmission electron microscopy. We found 145 and 160 virulence factors in the CA1 and CA2 strains, respectively. The extracellular CA1 strains grew faster than the CA2 and OG1RF strains, and the amount of intracellular viable bacteria in the OG1RF group was highest at 6 and 12 h post-infection. The macrophages in the CA1 infection group were the first to reach the maximum PI-positivity in the cell death time point curve. We found the expressions of mRNA expression of *caspase-1*, *GSDMD*, *caspase-3*, *MLKL*, *RIPK3*, *NLRP3*, *IL-1β* and *IL-18* and of proteins cleaved caspase-1, GSDMD, cleaved caspase-3 and pMIKL in the macrophages of the three infection groups to be upregulated (*P*<0.05). We detected ultrastructural changes of apoptosis, pyroptosis, and necroptosis in macrophages infected with *E. faecalis*. The three *E. faecalis* strains induced varying degrees of apoptosis, pyroptosis, and necroptosis that were probably associated with PANoptosis in macrophages. The *E. faecalis* CA1 strain exhibited faster growth and a higher real-time MOI, and it induced higher expression levels of some PANoptosis-related genes and proteins in the infected macrophages than the other strains tested.

## Introduction

Enterococci are ubiquitous in the digestive system of most complex metazoans, they belong to the commensal flora at approximately 10^4^ to 10^7^ bacteria per gram of feces. Despite its commensal nature, *Enterococcus faecalis* possesses virulence factors, such as lipoteichoic acid (major cell wall constituent), esp (protein surface), ace (collagen binding protein), gelE (gelatinase), and cylA (hemolysin activator), that make it an opportunistic pathogen related to hospital-acquired wound, urinary tract, and bloodstream infections (Kayaoglu and Orstavik, 2004; Wisplinghoff et al., 2004; Gjodsbol et al., 2006). *E. faecalis* is the most frequently isolated species in endodontic posttreatment teeth, with prevalence values reaching up to 90% of cases (Molander et al., 1998; Rocas et al., 2004; Stuart et al., 2006). *E. faecalis* continues to adapt to its environment, it possesses anti-starvation characteristics and can resist drugs and adverse environments (Gomes et al., 2004; Gomes et al., 2006; Sakko et al., 2016; Alghamdi and Shakir, 2020). *E. faecalis* can adhere to and invade cells, triggering or exacerbating inflammatory responses in periapical tissues.

The innate immune system detects microbial infections and activates programmed cell death (PCD) pathways, such as apoptosis, pyroptosis, and necroptosis. Research on microbial infections that cause cell death has mostly focused on the independent mechanism of a certain death pathway. Multiple lines of evidence indicate that a unified mechanism through common regulatory proteins and transmission molecules of the three signaling pathways, referred to as PANoptosis, plays a role in innate immune dysfunction during infectious diseases (Kuriakose and Kanneganti, 2019; Malireddi et al., 2020; Place et al., 2020). Moreover, researchers have explored the immune response of macrophages induced by *E. faecalis* pathogenic mechanisms in posttreatment periapical diseases (Chong et al., 2017). Zou et al. showed that *E. faecalis* infection may block apoptosis of macrophages by activating phosphatidylinositol 3-kinase signals (Zou and Shankar, 2014). *E. faecalis* induces pyroptosis in macrophages by activating the NLRP3 inflammasome, which leads to cleavage of caspase-1 and secretion of IL-1β (Yang et al., 2014; Ran et al., 2021). However, the study on the anti-infection response of macrophages induced by *E. faecalis* has focused mostly on a single PCD type. Whether macrophage death pathways exhibit multiple level crosstalk and can be activated simultaneously or consecutively within the same cell in posttreatment periapical diseases remains unclear.

In this study, we investigated dynamic changes in macrophages infected with either one of two viable root canal *E. faecalis* isolated strains or with a clinical oral isolate (OG1RF); we explored the real-time cell death of infected macrophages. We found that, after 6 and 12 h of real-time infection with viable *E. faecalis*, macrophages expressed genes and activated proteins (caspase-3 for apoptosis, NLRP3, gasdermin D [GSDMD] and caspase-1 for pyroptosis, and mixed lineage kinase domain-like protein [MLKL] and receptor interacting protein kinase 3 [RIPK3] for Necroptosis) that resulted in simultaneous occurrence of apoptosis, pyroptosis, and necroptosis of the macrophages. This is the first study documenting the simultaneous induction of apoptosis, pyroptosis, and necroptosis in macrophages infected with viable *E. faecalis*.

## Materials and Methods

### Bacterial and RAW264.7 Cell Cultures

Two *E. faecalis* isolated strains from retreated root canal samples and *E. faecalis* OG1RF were used in this study. The two isolated strains are called CA1 (CRISPR-Cas absence strain 1) and CA2 (CRISPR-Cas absence strain 2) due to their lack of a CRISPR-Cas module (Tong et al., 2017). We selected the *E. faecalis* OG1RF as a standard plasmid-free strain presenting the intrinsic CRISPR loci. Following routine streaking on brain heart infusion (BHI) agar (Difco, Becton Dickinson, Sparks, MD, USA) and aerobic culture, we inoculated a single bacterial colony into BHI broth to allow the bacteria to reach the exponential growth phase.

We cultured RAW264.7 murine macrophage line (ATCC, Manassas, VA) cells in alpha-minimal essential medium (α-MEM; Gibco, New York, NY, USA) with 10% fetal bovine serum (FBS; Gibco, New York, NY, USA) in a 5% CO_2_ humidified incubator at 37°C.

### Virulence Factor Annotation

The total DNAs of CA1 and CA2 strains were extracted using a TIANamp Bacterial DNA Kit (DP302, Tiangen Biotech, Beijing). The genomes of each strain were sequenced using an Illumina HiSeq 4000 system (Illumina, San Diego, CA, USA) at the Beijing Genomics Institute (Shenzhen, China), and we annotated the virulence factors of the strains based on data from the Virulence Factors of Pathogenic Bacteria database (VFDB, http://www.mgc.ac.cn/VFs/download.htm).

### RAW264.7 Cells Infected With *E. faecalis*

RAW264.7 cells were seeded overnight into 10-cm dishes at a density of 3×10^6^ cells/well for RT-qPCR, western blot, and transmission electron microscopy, onto 12-well culture plates at 3×10^4^ cells/well for determining *E. faecalis* and RAW264.7 cell viability, and into 96-well culture plates at 7×10^3^ cells/well for real-time cell death analysis. Macrophages were infected with one of each *E. faecalis* strains (CA1, CA2, and OG1RF groups) at a multiplicity of infection (MOI) of 100:1 and the cultures were incubated in a humidified environment with 5% CO_2_ at 37°C for a time dependent on the requirements of subsequent experiments.

### Quantification of *E. faecalis* and RAW264.7 Cells

Samples of RAW264.7 cells infected with either *E. faecalis* CA1, CA2, or OG1RF were placed in replicates onto the same two 12-well plates. One 12-well plate was used to quantify RAW264.7 macrophages and the other one to count bacteria. At 2, 6, and 12 h post-infection, we collected macrophages in 12-well plates using a cell scraper. Next, we applied a trypan blue stain to count viable cells in an automated cell counter (Cellometer Auto 1000, Nexcelom Bioscience, USA). We used uninfected RAW264.7 cells as controls. For counting extracellular bacteria, we transferred the bacterial suspensions and 3 PBS washouts per well into 15-ml Eppendorf tubes at 2, 6, or 12 h post-infection. Next, we decimally diluted the bacteria in the Eppendorf tubes and plated samples onto BHI agar plates. After 48 h of culture, we counted colony-forming units (CFUs) to quantify the extracellular bacteria in each tube. To count the intracellular bacteria, we incubated the RAW264.7 cells attached to the bottom of the 12-well plate with α-MEM plus 10% FBS containing niacin (10000 μg/mL) and ciprofloxacin (20 μg/mL) for 1 h to thoroughly kill any remaining extracellular bacteria (Xu et al., 2018). We plated samples on BHI agar after the 1-h incubation and confirmed the absence of bacterial growth and the efficacy of the treatment. RAW264.7 cells were lysed by adding a 1/10 volume of cell lysis solution to release the intracellular bacteria. We quantified these bacteria after plating decimally diluted samples and counting colony forming units. Raw CFUs were transformed to log_10_ values to normalize the data. The numbers of intracellular bacteria were recorded as CFUs per 10^4^ cells. We calculated real-time MOIs at 2, 6, and 12 h post-infection.

### Real-Time Cell Death Analysis

We performed real-time cell death analysis to explore dynamic changes in RAW264.7 cells in response to *E. faecalis* CA1, CA2 or OG1RF infection in 96-well plates cultured from 0 to 15 h. We used RAW264.7 cells suspended in 1% Triton for 15 min as the positive PI-staining control. RAW264.7 cells were stained with 0.5 μg/mL propidium iodide dye (PI, Solarbio, China) for 30 min, and we used a live cell imaging analysis system (BioTek, USA) to quantify the numbers of PI-positive cells in each well every half hour.

### Quantitative Real-Time Polymerase Chain Reaction for the Expression of PANoptosis-Related Genes

After being infected with *E. faecalis* CA1, CA2, or OG1RF at MOIs of 100:1 for 6 and 12 h, we washed RAW264.7 cells three times with PBS to remove floating dead cells. We extracted and purified total RNA samples from the viable RAW264.7 cells using an RNA-Quick purification kit (YISHAN Biotechnology, Shanghai, China). We used a Nanodrop 2000 spectrophotometer to quantify the purified RNA (Thermo Scientific, Waltham, MA, USA), and cDNA was synthesized using a reverse transcription kit (Takara, Kyoto, Japan). RT-qPCR was performed with SYBR qPCR Supermix Plus (Novoprotein, USA) on a QuantStudio 5 real-time PCR machine (Applied Biosystems, Thermo Fisher, USA). Relative expression levels of *caspase-3, caspase-1, GSDMD, MLKL, RIPK3, NLRP3, IL-1β*, and *IL 18* were normalized to the expression level of β-actin according to the 2^−ΔΔCt^ method. We searched the target gene mRNA sequence of mouse cells on the GenBank database, and the primers were designed using Primer express 5.0 software. The primer sequences used are listed in **Table 1**.

**Table 1:** Table of primer sequences for RT-qPCR.

Gene	Primers	Length	Annealing temperature	Gene function/Pathway
*Caspase 3*	Forward: 5‘-TGGCATTGAGACAGACAGTGG-3’ Reverse: 5‘-CCAGGAATAGTAACCAGGTGCTG-3’	109 Bp	60.27	Executioner caspase of apoptotic cell death
*RIPK3*	Forward: 5’-TCCACGCCAAGCTAGCAGAT-3’ Reverse: 5’-CCAGAGTCCCTGGATCCTGA-3’	91 Bp	60.03	Programmed Cell Death, RIPK1-mediated regulated necrosis, Regulated Necrosis, Regulation of necroptotic cell death, TRIF-mediated programmed cell death
*MLKL*	Forward: 5’-TGCAGAGGAAGACGGAAATGA-3’ Reverse: 5’-CTCCTGTGTGGGTTTTAGTGAGC-3’	112 Bp	59.38	Programmed Cell Death, RIPK1-mediated regulated necrosis, Regulated Necrosis, Regulation of necroptotic cell death
*NLRP3*	Forward: 5’-CTGAACCTGGGCAACAATGA-3’ Reverse: 5’-ACATTTCACCCAACTGTAGGCTC-3’	106 Bp	58.38	NLRP3 inflammasome
*Caspase 1*	Forward: 5‘-CCGAGGGTTGGAGCTCAAG-3’ Reverse: 5‘-TTCACCATCTCCAGAGCTGTGA-3’	107 Bp	60.08	Apoptosis, Programmed Cell Death, Pyroptosis, Regulated Necrosis
*GSDMD*	Forward: 5’- GAGCTTTATGCTTGAAGGGTGAA-3’ Reverse: 5’- CAGTTGGGCCACTCGGAAT-3’	101 Bp	59.24	Key effector leading to pyroptosis
*IL-1β*	Forward: 5’- CCAAAAGATGAAGGGCTGCTT-3’ Reverse: 5’- GAAAAGAAGGTGCTCATGTCCTC-3’	198Bp	59.10	Interleukin-1 signaling
*IL-18*	Forward: 5’- TGTCAGAAGACTCTTGCGTCAAC-3’ Reverse: 5’- GATTCCAGGTCTCCATTTTCTTCA-3’	91Bp	58.74	Interleukin-18 signaling
*β-actin*	Forward: 5’-GGCTGTATTCCCCTCCATCG-3’ Reverse: 5’-CCAGTTGGTAACAATGCCATGT-3’	154Bp	59.44	Reference gene

We searched the target gene mRNA sequence of mouse cells on the GenBank database, and designed specific primers using Primer express 5.0 software.

### Western Blot for the Expression of PANoptosis-Related Proteins

After being infected with *E. faecalis* CA1, CA2, or OG1RF, RAW264.7 cells were washed three times with PBS to remove floating dead cells. We extracted total protein samples from the RAW264.7 cells harvested at 6 and 12 h after infection and used a BCA protein assay kit (Beyotime, China) following the manufacturer’s instructions to quantify the total proteins in each sample. Proteins were separated on a 12% SurePAGE precast gel and electro-transferred to PVDF membranes. After blocking in 5% nonfat milk at room temperature for 1 h, the membranes were incubated with primary antibodies overnight at 4°C. We used antibodies against PANoptosis-related proteins and controls including ß-actin (1:1000, Cell Signaling Technology [CST], #4970), caspase-3 (1:1000, CST, #9662), caspase-1 (1:1000, CST, #89332), GSDMD (1:1000, Abcam, ab209845), pMLKL(1:1000, CST, #37333), and MLKL(1:1000, CST, #37705). Subsequently, membranes were incubated with horseradish peroxidase (HRP)-conjugated goat anti-rabbit secondary antibody (1:5000) for 1 h at room temperature. We visualized protein bands using an ECL kit (Millipore, Bedford, MA, USA) under the Image Quant LAS 4000 mini imaging system. Relative protein levels were quantified by analyzing the scanned protein bands in the ImageJ software and normalizing intensity values to that of the β-actin band on the same blotting membrane.

### Transmission Electron Microscopy

We detected ultrastructural changes in RAW264.7 cells infected with *E. faecalis* OG1RF by transmission electron microscopy (TEM). We rinsed RAW264.7 cells infected with *E. faecalis* for 6 and 12 h with PBS in a 10-cm dish after discarding the supernatant. Next, we collected the RAW264.7 cells using a scraper and fixed the cells with 2.5% glutaraldehyde and 2% sodium formate. After centrifugation (500×g/min for 15 min), we rinsed the cells with arsenate buffer, soaked them in a 0.1 M sodium cacodylate solution containing 1% osmium tetroxide and 0.5% potassium ferrocyanide at room temperature for 2 h, dehydrated them in a series of acetone solutions, dried them under vacuum, embedded them in an Epon 812 epoxy resin, and made 50-nm ultrathin sections before double staining them with acetic acid, dioxygen axis, and lead citrate, and observing them under TEM (Tecnai G2 SpiritTwin, FEI, Czech Republic).

### Statistical Analysis

All experiments were repeated at least three times. We expressed data as means ± standard deviations (SDs) using SPSS 25.0. Data conforming to the normal distribution and homogeneity of variance were compared among multiple groups using one-way ANOVA, followed by Tukey’s *post hoc* test. We used Kruskal-Wallis tests for data with uneven variance. The level of significance was set at *P*<0.05.

## Results

### Annotation of the Two Root Canal *E. faecalis* Isolated Strains in the VFDB Database

We identified 145 and 160 pathogenic virulence factor genes in the CA1 and CA2 strains, respectively, using the gene functional annotation feature of the VFDB database. The CA1 and CA2 strains contained 28 and 37 capsule production-related genes, respectively. Among these genes, *cpsB-K* is derived from *E. faecalis* V583. In addition, 9 genes in the CA1 strain, including endocarditis-biofilm fimbriae (*Ebp pili*) and a hyaluronidase, were absent in the CA2 strain. Meanwhile, 24 genes in strain CA2, including 12 capsular polysaccharide-related virulence genes, were not identical to those in the CA1 strain (**Supplementary Tables 1** and **2**).

### Viability of Extracellular and Intracellular Bacteria and Real-Time MOI Changes

**Figure 1:**
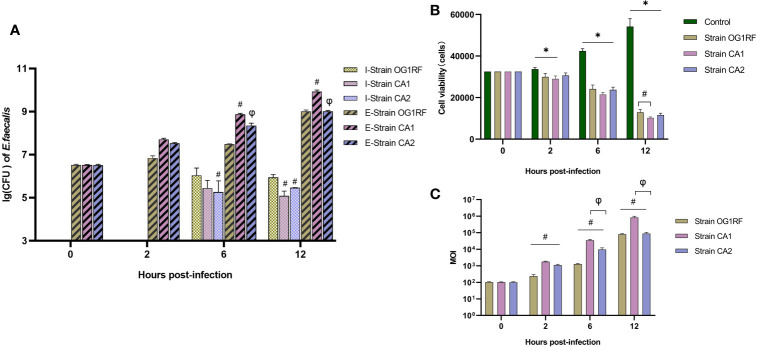
Viability of extracellular and intracellular bacteria and real-time MOI changes in co-culture of *E. faecalis* and RAW264.7 cells. RAW264.7 cells seeded overnight in 12-well culture plates at 3×10^4^/well were infected with the *E. faecalis* CA1, CA2, or OG1RF strains at a multiplicity of infection (MOI) of 100:1. Extracellular and intracellular bacteria and RAW264.7 cells were counted at 2, 6, and 12 h of infection. **(A)** Viability of intracellular (I-strains) and extracellular (E-strains) bacteria [lg (CFU/10^4^ cells)]. **(B)** Viability of RAW264.7 cells. **(C)** Real-time MOI of co-culture of *E. faecalis* and RAW264.7 cells. *P < 0.05, compared to the control group. ^#^ *P < *0.05, compared to the OG1RF group, ^φ^ *P* < 0.05 compared to the CA1 group.

Our quantification of extracellular bacteria showed that the CA1 strain grew more rapidly than either the CA2 or the OG1RF strains (*P*<0.05), which both grew at similar rates (*P*>0.05). We found no intracellular bacteria at 2 h post-infection in any of the three infected macrophage groups; however, we found viable bacteria at both 6 and 12 h post-infection. The CFUs of intracellular viable bacteria in the OG1RF group were more abundant than those of the CA1 and CA2 groups at 12 h post-infection (*P*<0.05) (**Figure 1A**). The numbers of viable RAW264.7 cells in the three *E. faecalis* infection groups were significantly lower than those in the control group. At 12 h post-infection, the number of viable macrophages with OG1RF infection was the highest, followed by those with CA2 and CA1 infections (**Figure 1B**). At 2 h post-infection, the real-time MOIs in the CA1 and CA2 groups were higher than that in the OG1RF group (*P*<0.05); but, at 6 and 12 h, the real-time MOI in the CA1 group was higher than those in the OG1RF and CA2 groups (*P*<0.05) (**Figure 1C**).

### Dynamic Changes in Macrophages Infected With *E. faecalis*

**Figure 2:**
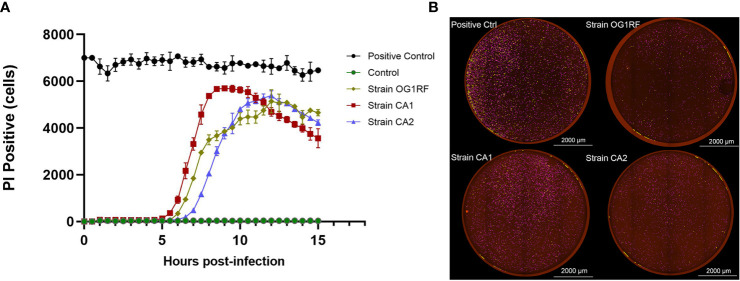
Quantification of PI-positivity over time in RAW264.7 cell cultures infected with each of the three strains of *E. faecalis* (MOI = 100:1). **(A)** Live cell imaging (4×) quantification of PI-positivity in RAW264.7 cells infected with each of the three *E. faecalis* strains. **(B)** Representative cell death images with the red mask indicating dead cells at the maximum PI-positivity value. Scale bar: 2000 μm.

In the real-time cell death analysis of RAW264.7 macrophages infected with *E. faecalis* CA1, CA2, and OG1RF strains, PI-positive cells started to become apparent at 5 to 6 h post-infection. In the CA1 group, PI-positivity reached the maximum at 8.5 h, followed by those of CA2 and OG1RF strains at 12 h (**Figure 2**).

### *E. faecalis* Infection Upregulated PANoptosis-Related Genes and Proteins in Macrophages

**Figure 3:**
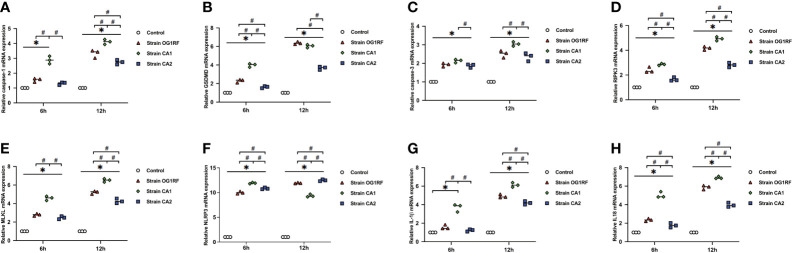
Relative expression of PANoptosis-related genes in RAW264.7 cells at 6 and 12 h after *E. faecalis* infection by RT-qPCR. **(A)** *caspase-1*, **(B)** *GSDMD*, **(C)** *caspase-3*, **(D)** *RIPK3*, **(E)** *MLKL*, **(F)** *NLRP3*, **(G)** *IL-1β*, and **(H)** *IL-18.* All experiments were repeated three times. *P < 0.05 compared to the control group. ^#^ *P* < 0.05 compared to other experimental groups.

**Figure 4:**
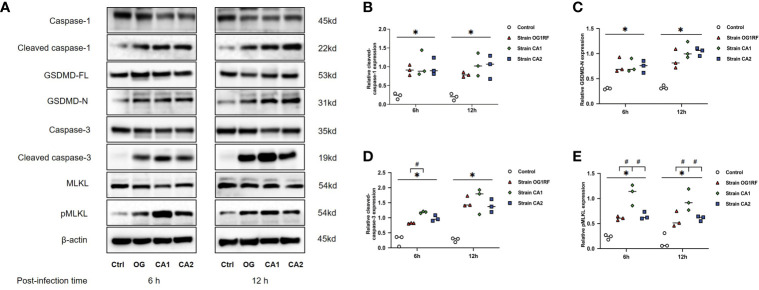
Western blots showing the relative expression of PANoptosis-related proteins in RAW264.7 cells 6 and 12 h after *E. faecalis* infection. **(A)** Representative immunoblotting bands of eight proteins. Quantification of cleaved caspase-1 **(B)**, cleaved caspase-3 **(C)**, GSDMD-N **(D)**, and pMLKL **(E)**. Relative protein levels were quantified by analyzing the scanned protein bands using the ImageJ software after normalization to β-actin levels on the same blots. All experiments were repeated three times. *P < 0.05 compared to the control group. ^#^ *P* < 0.05 compared to the other experimental groups.

At 6 and 12 h post-infection, the mRNA expression levels of *caspase-3*, *caspase-1*, *GSDMD*, *RIPK3*, *MLKL*, *NLRP3*, *IL-1β*, and *IL-18* were upregulated in RAW264.7 macrophages infected with either *E. faecalis* CA1, CA2, or OG1RF strains compared to the levels in the control group. The eight PANoptosis-related genes exhibited the highest expression in macrophages infected with CA1, except for *NLRP3* and *GSDMD* at 12 h (**Figure 3**). Apoptosis is associated with expression of cleaved caspase-3; necroptosis with expression of pMLKL; and pyroptosis with expression of cleaved caspase-1 and GSDMD-N in macrophages. Similarly, we found upregulated expressions of cleaved caspase-3, pMLKL, cleaved caspase-1, and GSDMD-N proteins in the three *E. faecalis* infection groups at 6 and 12 h post-infection, compared to the protein expression levels in the control group (*P* < 0.05). The pMLKL protein was more highly expressed in the CA1 infection group than in either of the other infection groups at 6 and 12 h (**Figure 4**).

### Ultrastructural Changes in Macrophages Infected With *E. faecalis*

**Figure 5:**
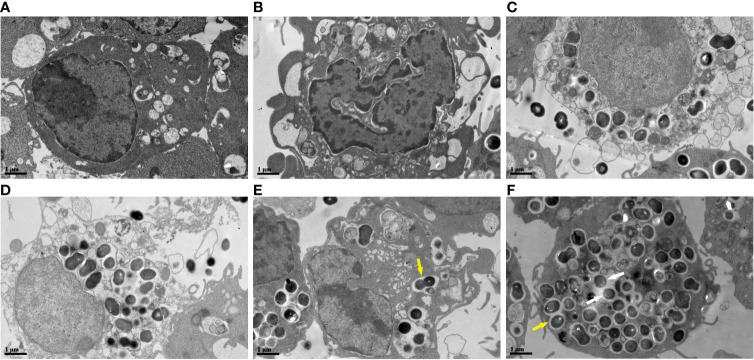
Ultrastructural changes of RAW264.7 cells infected with *E. faecalis* OG1RF under TEM **(A)** Control RAW264.7 cells showing an intact cell membrane. **(B)** Cellular ultrastructure of apoptotic-like changes: cell shrinkage. **(C)** Cellular ultrastructure of pyroptotic-like changes: swollen cells with incomplete cell membrane. **(D)** Cellular ultrastructure of necroptotic-like changes: swollen cells with ruptured cell membrane. **(E)** Engulfed *E. faecalis* in monolayer membrane vesicles 6 h post-infection. **(F)** Some escaped *E. faecalis* cells outside of membrane vesicles. The yellow arrow indicates *E. faecalis* surrounded by monolayer membrane vesicles, and the white arrow represents escaped *E. faecalis* cells outside of membrane vesicles. Scale bar: 1μm.

The cellular ultrastructure of the uninfected macrophages exhibited intact cell membranes and normal cell organelles structures, such as Golgi bodies, rough endoplasmic reticulum, and mitochondria (**Figure 5A**). We found PANoptosis-related ultrastructural changes in the infected macrophages. Apoptosis-like cells displayed cell shrinkage, crescent-like nuclear deformation, hyperchromatic nuclei, and many mitochondria around the nucleus (**Figure 5B**). The suspected pyroptosis-like cells presented swollen cell bodies, incomplete cell membranes, deformed nuclear membranes, and damaged mitochondrial and lipid vacuoles (**Figure 5C**). Moreover, some suspected necroptosis-like cells exhibited significantly swollen cell bodies, ruptured cell membranes, cellular content effusions, and many lipid vacuoles (**Figure 5D**). In macrophages after 6 h of infection, most *E. faecalis* bacteria were enclosed by monolayer membrane vesicles (**Figure 5E**). The abundance of macrophage-engulfed *E. faecalis* bacteria was higher at 12 h post-infection, and some bacteria escaped from membrane vesicles, indicating resistance to macrophage killing and the capability to survive within the cytoplasm for extended periods (**Figure 5F**).

## Discussion

Some microorganisms have evolved strategies to evade the inflammatory mechanisms of immune cells; however, host cells have also evolved complex response strategies. Multiple interconnected mechanisms sense abnormalities in key protein signaling pathways and initiate the assembly of a variety of cell death complex PANoptosomes to induce PCD and prevent infections (Mukherjee et al., 2006; Paquette et al., 2012; Philip et al., 2014; Orning et al., 2018; Sanjo et al., 2019). The three PANoptosis-related PCD types (apoptosis, pyroptosis and necroptosis) are regulated by the PANoptosome complex (Christgen et al., 2020; Malireddi et al., 2020; Place et al., 2020). PANoptosis is critical for restricting a wide range of pathogens, such as bacteria, viruses, fungi, and parasites (Place et al., 2020). Whether PANoptosis occurs in macrophages infected with *E. faecalis* was uncertain when we designed this study. We first evaluated the real-time cell death of *E. faecalis* infected macrophages and found significant upregulation of the three PANoptosome effectors (cleavage of caspase-3, pMLKL and GSDMD-N proteins) at 6 and 12 h post-infection. Moreover, a few macrophages infected with *E. faecalis* had ultrastructural changes typical of apoptosis, pyroptosis, or necroptosis. These results suggest that PANoptosis may occur in macrophages infected with a high concentration of viable *E. faecalis*.

Different numbers and types of pathogenic bacteria caused cell death differences in our study. We initially used 100:1 MOIs to evaluate programmed cell death in macrophages, but the real-time MOIs reached 10^3^ to 10^4^ after 6 h and 10^5^ to 10^6^ after 12 h of infection due to bacterial growth and macrophage death. *Yersinia*, *Salmonella enterica*, *Enteropathogenic Escherichia coli*, *Listeria monocytogenes*, and *Francisella novicida* infections may activate multiple cell death processes *via* PANoptosis that prevent pathogens from evading detection (Christgen et al., 2020; Lacey and Miao, 2020; Place et al., 2020). Paquette et al. found that *Yersinia* inhibits TAK1 in infected mouse bone marrow macrophages (BMDMs), a feature that may help the bacteria to evade the host immune response (Paquette et al., 2012). However, the infected BMDMs responded with the activation of the intracellular PANoptosome complex (composed of RIPK1, caspase-8, ASC, and NLRP3 activated caspase-3/-7), the promotion of GSDMD phosphorylation and cleavage by MLKL, the induction of PANoptosis, and the released of IL-1β and IL-18. Similarly, in another study, Christgen et al. found that macrophages infected with *influenza A virus*, *vesicular stomatitis virus*, *Listeria monocytogenes*, or *Salmonella enterica serovar Typhimurium* activated PANoptosis mechanisms, resulting in massive cell death (Christgen et al., 2020). These results suggest that the simultaneous activation of pyrolysis, apoptosis, and necroptosis is an important immune mechanism for macrophages to respond to microbial infections. Cell death may protect the organism against most acute bacterial pathogens. We found no evidence for *E. faecalis-*related PANoptosis in macrophages in the literature. In our study, high concentrations of *E. faecalis* simultaneously induced apoptosis, pyroptosis, and necroptosis in macrophages, suggesting the activation of different cell death pathways that may be related to PANoptosis in macrophages and that warrant further exploration of the phenomenon.

Pyroptosis and necroptosis are characterized as lytic forms of cell death driven by activation of pore-forming proteins, while apoptosis maintains tissue homeostasis by eliminating aged or damaged cells and serves as an important infection control mechanism (Green et al., 2009). In a study by Zou et al, 1 h of *E. faecalis* E99 strain (a clinical isolate from the urine of a patient) infection at an MOI of 10:1 prevented apoptosis in macrophages subjected to a wide spectrum of proapoptotic stimuli (Zou and Shankar, 2014). In Mohamed Elashiry et al’s study, a low *E. faecalis* MOI (1:1) in bone marrow stem cells (BMSCs) for a prolonged time reduced the apoptotic activity in subsequently differentiated macrophages (Mohamed Elashiry et al., 2021). The results of the two studies indicate that *E. faecalis* at low MOIs represses apoptosis of macrophages and decreases apoptotic cell deaths to facilitate a continuous infection. Similarly, we found no PI-positive macrophages before 5 h of infection in our study, indicating the absence of dead cells. However, our experiments showed that the three types of *E. faecalis* infection induced apoptosis in macrophages when the bacterial proliferation reached a high concentration. At 6 h post-infection, *E. faecalis* induced significant upregulation of caspase-3 gene and protein expressions, and a few *E. faecalis* were engulfed. Thus, we infer that a few *E. faecalis* bacteria may survive within macrophages for extended periods of time by inhibiting apoptosis and promoting infection spread. By contrast, a high concentration of *E. faecalis* bacteria expressing many virulence factors was associated with apoptosis, pyroptosis, and necroptosis. Compared to the events in CA2 and OG1RF strains at equivalent time points, the CA1 strain grew faster in co-culture with RAW264.7 macrophages, and it reached a higher real-time MOI and led to a more pronounced macrophage destruction. This may explain the fast PI-positivity of macrophages infected with the CA1 strain and the high expression of some PANoptosis-related genes and proteins at 6 and 12 h post-infection in the CA1 group. These results show differences in the effects of different bacterial strains of the same species on the PCD of macrophages.

In our study, we did not detect viable *E. faecalis* bacteria within macrophages at 2 h post-infection. In a study by Xu et al, live *E. faecalis* infection at an MOI of 100:1 for 2 h revealed no cytotoxic effect on RAW264.7 cells, but it improved the metabolic and phagocytosis activity of macrophages (Xu et al., 2018). We first detected intracellular bacteria at 6 h post-infection, and their abundance increased at 12 h post-infection. The existence of viable intracellular bacteria implies the survival of *E. faecalis* in macrophages, a fact that we confirmed by the presence of intracytoplasmic bacteria under TEM. Viable *E. faecalis* survival in macrophages was also found in the studies of Sabatino et al. and Zou et al. (Sabatino et al., 2015; Zou and Shankar, 2016). In our comparison of the three different *E. faecalis* strain infections, at 12 h post-infection (real-time MOI > 10^5^), the numbers of intracellular viable bacteria in CA1- and CA2-infected macrophages were significantly lower than that in the OG1RF-infected macrophages. According to the functional annotation of our two root canal isolated strains’ genes in the VFDB database, many virulence factor genes originated from other bacterial genera, and this finding may be associated with the absence of the CRISPR-cas system and a strong acquisition of exogenous virulence genes (Burley and Sedgley, 2012; Hullahalli et al., 2018; Gholizadeh et al., 2020). These VFDB gene functional annotations may explain immunity escape mechanisms in these strains. *E. faecalis* CA1 and CA2 strains possess *cpsB-K* genes derived from *E. faecalis* V583; in particular, *cpsC-E, cpsG*, and *cpsI-K* are closely related to capsule formation (**Supplementary Tables 1** and **2**). These genes help bacteria to avoid immune recognition, phagocytosis, and clearance (Hancock and Gilmore, 2002; Thurlow et al., 2009a; Thurlow et al., 2009b). The *E. faecalis* OG1RF strain with its intrinsic CRISPR loci and devoid of any plasmids may not possess many exogenous genes, and it cannot escape macrophage phagocytosis.

Despite their lack of a CRISPR-cas system, CA1 and CA2 strains presented distinct virulence factor genes. Each *E. faecalis* strain survives in their individual clinical environment and acquires different exogenous genes from the other bacteria. Our functional gene annotation in the VFDB database revealed 9 virulence factor genes in the CA1 strain absent from the CA2 strain. These 9 virulence factor genes might explain the fast infection proliferation of the CA1 strain. Most virulence factor genes in the CA2 strain were related to capsule formation, which may enhance bacterial resistance to host cell immune clearance (Hancock and Gilmore, 2002; Thurlow et al., 2009a; Thurlow et al., 2009b). These differences between the root canal isolates, may explain why the PI-positivity of macrophages infected with the CA2 strain appeared after those of the other infected macrophages, and the relatively lower expression of PANoptosis-related genes in the CA2 strain infection group than in the OG1RF strain infection. These results suggest that the pathogenic features of the CA2 strain may not cause excessive cell death, but promote immune escape. Immune escape mechanisms allow pathogenic bacteria to generate persistent infections. Notwithstanding, PI-positive CA2- and OG1RF-infected macrophages ended up reaching similar values, suggesting that the PANoptosis in macrophages infected with *E. faecalis* may target these immune escaping pathogens. Therefore, studying the mechanisms of PANoptosis in *E. faecalis*-infected macrophages will help us understand the pathogenesis of periapical inflammation.

In this study, we used three *E. faecalis* strains associated with PCD apoptosis, pyroptosis, or necroptosis in macrophages, which suggests a possible role for PANoptosis in periapical infections. More than 460 unique bacterial taxa (belonging to 100 genera and 9 phyla) have been detected in various root canal infections with apical periodontitis (Siqueira and Rôças, 2009), and these polymicrobial infections complicate the study of immune regulation of PCD by the immune cells.

## Conclusions

Within the limitations of this *in vitro* study, two *E. faecalis* root canal-isolated strains and the OG1RF strain induced apoptosis, pyroptosis, and necroptosis in macrophages 6 and 12 h after real-time infection. We found viable intracellular bacteria in macrophages infected with *E. faecalis* for 6 and 12 h. The root canal-isolated CA1 strain showed faster growth, a higher real-time MOI, and higher expression of many PANoptosis-related genes and proteins than the other *E. faecalis* strains infecting macrophages.

## Data Availability Statement

The original contributions presented in the study are included in the article/**Supplementary Material**. Further inquiries can be directed to the corresponding author.

## Author Contributions

DC, XL, and ZT conceptualized the study. DC, XL, QM, JT, QG, and ZT designed the methodology and conducted the analysis. DC, XL, and QM performed the experiments. DC, XL, and ZT wrote the manuscript with input from all the authors. JT, QG, and ZT acquired the funding and provided overall supervision. All authors contributed to the article and approved the submitted version.

## Funding

This study was supported by the National Natural Science Foundation of China (grant number 81870750) and Science and Technology Planning Project of Guangdong Province, China (2020A0505100034).

## Conflict of Interest

The authors declare that the research was conducted in the absence of any commercial or financial relationships that could be construed as a potential conflict of interest.

## Publisher’s Note

All claims expressed in this article are solely those of the authors and do not necessarily represent those of their affiliated organizations, or those of the publisher, the editors and the reviewers. Any product that may be evaluated in this article, or claim that may be made by its manufacturer, is not guaranteed or endorsed by the publisher.
