# Supplementary material for: Real-Time Induction of Macrophage Apoptosis, Pyroptosis, and Necroptosis by Enterococcus faecalis OG1RF and Two Root Canal Isolated Strains
Source: Front Cell Infect Microbiol. 2021 Aug 26;11:720147. doi: 10.3389/fcimb.2021.720147 (PMC8427696; doi:10.3389/fcimb.2021.720147)
Supplement: Supplementary file 2 [file Table_2.docx]

**Table 2 The virulence factors of CA2 found in the VEDB database**

| **Gene_id** | **Identity** | **E_value** | **VF_id** | **GI_id** | **Type** | **Description** |
| --- | --- | --- | --- | --- | --- | --- |
| CA2GL002446 | 40.2 | 4.40E-33 | VFG035924 | gi:387610457 | Predicted | (aatC) ABC transporter ATP-binding protein AatC [ABC transporter for dispersin (CVF737)] [Escherichia coli O78:H11:K80 str. H10407] |
| **CA2GL001201** | **70.7** | **4.80E-151** | **VFG037994** | **gi:387125713** | **Predicted** | **(ABTJ_03752) UDP-N-acetylglucosamine 2-epimerase [Capsule (CVF775)] [Acinetobacter baumannii MDR-TJ]** |
| CA2GL001083 | 86.8 | 1.20E-265 | VFG002166 | gb\|NP_814829 | Verified | (ace) collagen adhesin protein [Ace (VF0355)] [Enterococcus faecalis V583] |
| CA2GL001236 | 45.1 | 4.10E-12 | VFG011430 | gb\|NP_540392 | Verified | (acpXL) acyl carrier protein [LPS (CVF383)] [Brucella melitensis bv. 1 str. 16M] |
| **CA2GL001241** | **46.3** | **1.70E-56** | **VFG032679** | **gi:347547558** | **Predicted** | **(agrA) putative 2-components response regulator protein [AgrA/AgrC (CVF254)] [Listeria ivanovii subsp. ivanovii PAM** |
| CA2GL000305 | 54.3 | 4.50E-143 | VFG049120 | gb\|YP_002918207.1 | Verified | (allB) allantoinase [Allantion utilization (VF0572)] [Klebsiella pneumoniae subsp. pneumoniae NTUH-K2044] |
| CA2GL000304 | 56.7 | 1.10E-129 | VFG049122 | gb\|YP_002918211.1 | Verified | (allC) allantoate amidohydrolase [Allantion utilization (VF0572)] [Klebsiella pneumoniae subsp. pneumoniae NTUH-K2044] |
| CA2GL002738 | 53.9 | 1.40E-108 | VFG049124 | gb\|YP_002918212.1 | Verified | (allD) ureidoglycolate dehydrogenase [Allantion utilization (VF0572)] [Klebsiella pneumoniae subsp. pneumoniae NTUH-K2044] |
| CA2GL001443 | 47.2 | 6.90E-80 | VFG015903 | gi:71735587 | Predicted | (argK) phaseolotoxin-insensitive ornithine carbamoyltransferase [Phytotoxin phaseolotoxin (CVF546)] [Pseudomonas syringae pv. phaseolicola 1448A] |
| **CA2GL001192** | **49.6** | **2.00E-59** | **VFG016435** | **gi:118480319** | **Predicted** | **(BALH_4781) tyrosine-protein kinase [Polysaccharide capsule (CVF567)] [Bacillus thuringiensis str. Al Hakam]** |
| CA2GL000809 | 42.9 | 4.10E-68 | VFG016372 | gi:30023297 | Predicted | (BC5265) membrane-bound transcriptional regulator LytR [Polysaccharide capsule (CVF567)] [Bacillus cereus ATCC 14579] |
| **CA2GL001193** | **47.1** | **1.70E-62** | **VFG016383** | **gi:30023308** | **Predicted** | **(BC5276) Phosphotyrosine-protein phosphatase (capsular polysaccharide biosynthesis) [Polysaccharide capsule (CVF567)] [Bacillus cereus ATCC 14579]** |
| **CA2GL001196** | **72.1** | **1.10E-155** | **VFG016400** | **gi:42784439** | **Predicted** | **(BCE_5394) aminotransferase family protein [Polysaccharide capsule (CVF567)] [Bacillus cereus ATCC 10987]** |
| CA2GL000906 | 47.6 | 6.00E-142 | VFG026307 | gi:116688795 | Predicted | (Bcen2424_0772) glycosyl transferase family protein [Capsule I (CVF645)] [Burkholderia cenocepacia HI2424] |
| **CA2GL002534** | **74.4** | **1.30E-147** | **VFG019385** | **gi:226224673** | **Predicted** | **(bsh) conjugated bile acid hydrolase [Bile-salt hydrolase (CVF250)] [Listeria monocytogenes serotype 4b str. CLIP 80459]** |
| **CA2GL001200** | **59.9** | **6.70E-129** | **VFG001302** | **gb\|NP_644944** | **Verified** | **(cap8F) capsular polysaccharide synthesis enzyme Cap8F [Capsule (VF0003)] [Staphylococcus aureus subsp. aureus MW2]** |
| CA2GL002478 | 42.9 | 2.90E-12 | VFG005069 | gi:49482401 | Predicted | (cap8J) capsular polysaccharide synthesis enzyme [Capsule (CVF110)] [Staphylococcus aureus subsp. aureus MRSA252] |
| **CA2GL001194** | **55.8** | **7.60E-175** | **VFG006072** | **gi:125719003** | **Predicted** | **(capD) Cps9E, putative [Capsule (CVF186)] [Streptococcus sanguinis SK36]** |
| **CA2GL001199** | **76** | **5.30E-149** | **VFG005020** | **gi:70725393** | **Predicted** | **(capE) capsular polysaccharide synthesis enzyme CapE [Capsule (CVF110)] [Staphylococcus haemolyticus JCSC1435]** |
| CA2GL001470 | 42.3 | 1.90E-44 | VFG039536 | gb\|NP_820549 | Verified | (CBU_1566) Coxiella Dot/Icm type IVB secretion system translocated effector [T4SS effectors (CVF803)] [Coxiella burnetii RSA 493] |
| CA2GL002578 | 46.5 | 7.60E-57 | VFG039536 | gb\|NP_820549 | Verified | (CBU_1566) Coxiella Dot/Icm type IVB secretion system translocated effector [T4SS effectors (CVF803)] [Coxiella burnetii RSA 493] |
| CA2GL000862 | 42.3 | 7.20E-21 | VFG039488 | gi:212218227 | Predicted | (CbuK_0601) hypothetical protein [T4SS effectors (CVF803)] [Coxiella burnetii CbuK_Q154] |
| CA2GL001596 | 59.9 | 5.40E-288 | VFG000079 | gb\|NP_463763 | Verified | (clpC) endopeptidase Clp ATP-binding chain C [ClpC (VF0072)] [Listeria monocytogenes EGD-e] |
| CA2GL002542 | 48.1 | 9.00E-167 | VFG000080 | gb\|NP_464522 | Verified | (clpE) ATP-dependent protease [ClpE (VF0073)] [Listeria monocytogenes EGD-e] |
| **CA2GL002621** | **61.4** | **1.60E-251** | **VFG000080** | **gb\|NP_464522** | **Verified** | **(clpE) ATP-dependent protease [ClpE (VF0073)] [Listeria monocytogenes EGD-e]** |
| CA2GL001943 | 81.6 | 3.60E-89 | VFG000077 | gb\|NP_465991 | Verified | (clpP) ATP-dependent Clp protease proteolytic subunit [ClpP (VF0074)] [Listeria monocytogenes EGD-e] |
| CA2GL001783 | 57 | 1.40E-116 | VFG001373 | gb\|NP_344890 | Verified | (cps4I) UDP-N-acetylglucosamine-2-epimerase [Capsule (VF0144)] [Streptococcus pneumoniae TIGR4] |
| **CA2GL000425** | **100** | **1.00E-155** | **VFG002190** | **gb\|NP_816141** | **Verified** | **(cpsA) undecaprenyl diphosphate synthase [Capsule (VF0361)] [Enterococcus faecalis V583]** |
| CA2GL000424 | 100 | 4.60E-148 | VFG002189 | gb\|NP_816140 | Verified | (cpsB) phosphatidate cytidylyltransferase [Capsule (VF0361)] [Enterococcus faecalis V583] |
| CA2GL002474 | 100 | 1.30E-231 | VFG002188 | gb\|NP_816139 | Verified | (cpsC) teichoic acid biosynthesis protein, putative [Capsule (VF0361)] [Enterococcus faecalis V583] |
| CA2GL002473 | 99 | 1.60E-287 | VFG002187 | gb\|NP_816138 | Verified | (cpsD) glycosyl transferase, group 2 family protein [Capsule (VF0361)] [Enterococcus faecalis V583] |
| CA2GL002472 | 99.5 | 0.00E+00 | VFG002186 | gb\|NP_816137 | Verified | (cpsE) glycosyl transferase, group 2 family protein [Capsule (VF0361)] [Enterococcus faecalis V583] |
| CA2GL002471 | 100 | 0.00E+00 | VFG002184 | gb\|NP_816135 | Verified | (cpsG) MurB family protein [Capsule (VF0361)] [Enterococcus faecalis V583] |
| CA2GL002470 | 97.6 | 7.10E-67 | VFG002183 | gb\|NP_816134 | Verified | (cpsH) lipoprotein [Capsule (VF0361)] [Enterococcus faecalis V583] |
| CA2GL002469 | 100 | 4.20E-199 | VFG002182 | gb\|NP_816133 | Verified | (cpsI) UDP-galactopyranose mutase [Capsule (VF0361)] [Enterococcus faecalis V583] |
| CA2GL002468 | 98.4 | 2.70E-249 | VFG002181 | gb\|NP_816132 | Verified | (cpsJ) ABC transporter, ATP-binding protein [Capsule (VF0361)] [Enterococcus faecalis V583] |
| CA2GL002467 | 100 | 1.10E-152 | VFG002180 | gb\|NP_816131 | Verified | (cpsK) ABC transporter, permease protein [Capsule (VF0361)] [Enterococcus faecalis V583] |
| CA2GL001569 | 60.3 | 1.00E-198 | VFG043573 | gi:15605121 | Predicted | (CT396) molecular chaperone DnaK [MOMP (AI392)] [Chlamydia trachomatis D/UW-3/CX] |
| CA2GL001719 | 54 | 4.30E-77 | VFG012174 | gi:125973345 | Predicted | (Cthe_0827) hemolysin A [Hemolysin (CVF417)] [Clostridium thermocellum ATCC 27405] |
| CA2GL002770 | 47.6 | 2.00E-165 | VFG031407 | gi:433630073 | Predicted | (ctpV) Putative metal cation transporter P-type ATPase CtpV [Copper exporter (CVF658)] [Mycobacterium canettii CIPT 140070010] |
| CA2GL001299 | 50.2 | 7.40E-139 | VFG032791 | gi:116872374 | Predicted | (dltA) D-alanine--poly(phosphoribitol) ligase subunit 1 [D-alanine-polyphosphoribitol ligase (CVF676)] [Listeria welshimeri serovar 6b str. SLCC5334] |
| CA2GL000241 | 99.6 | 0.00E+00 | VFG002195 | gb\|NP_814561 | Verified | (EF0818) polysaccharide lyase, family 8 [Hyaluronidase (VF0359)] [Enterococcus faecalis V583] |
| CA2GL001885 | 99.4 | 2.10E-176 | VFG002165 | gb\|NP_815739 | Verified | (efaA) endocarditis specific antigen [EfaA (VF0354)] [Enterococcus faecalis V583] |
| CA2GL001258 | 99.7 | 3.60E-190 | VFG045668 | gi:397699344 | Predicted | (EFD32_0765) bacterial regulatory protein, LacI family protein [BopD (CVF615)] [Enterococcus faecalis D32] |
| CA2GL001650 | 99.6 | 0.00E+00 | VFG045637 | gi:397699478 | Predicted | (EFD32_0899) Endocarditis and Biofilm-Associated Pilus subunitA [Ebp pili (CVF821)] [Enterococcus faecalis D32] |
| CA2GL001649 | 99.6 | 3.30E-266 | VFG045643 | gi:397699479 | Predicted | (EFD32_0900) Endocarditis and Biofilm-Associated Pilus subunitB [Ebp pili (CVF821)] [Enterococcus faecalis D32] |
| CA2GL000334 | 91.7 | 0.00E+00 | VFG045666 | gi:397701177 | Predicted | (EFD32_2606) polysaccharide lyase family protein [Hyaluronidase (CVF614)] [Enterococcus faecalis D32] |
| CA2GL001831 | 82.7 | 3.80E-200 | VFG005579 | gi:116516768 | Predicted | (eno) phosphopyruvate hydratase [Streptococcal enolase (CVF153)] [Streptococcus pneumoniae D39] |
| CA2GL000373 | 40.2 | 9.70E-77 | VFG047736 | unknown | Predicted | (F7308_0022) carbamoyl-phosphate synthase small chain [Pyrimidine biosynthesis (CVF845)] [Francisella sp. TX077308] |
| **CA2GL000920** | **42.5** | **4.10E-74** | **VFG046913** | **unknown** | **Predicted** | **(F7308_0918) glycosyl transferase [LPS (CVF834)] [Francisella sp. TX077308]** |
| **CA2GL001793** | **40.5** | **5.50E-68** | **VFG046913** | **unknown** | **Predicted** | **(F7308_0918) glycosyl transferase [LPS (CVF834)] [Francisella sp. TX077308]** |
| CA2GL001482 | 48.3 | 6.00E-30 | VFG011402 | gi:23502030 | Predicted | (fabZ) (3R)-hydroxymyristoyl ACP dehydratase [LPS (CVF383)] [Brucella suis 1330] |
| CA2GL002784 | 41.8 | 6.20E-22 | VFG011402 | gi:23502030 | Predicted | (fabZ) (3R)-hydroxymyristoyl ACP dehydratase [LPS (CVF383)] [Brucella suis 1330] |
| CA2GL001407 | 45.6 | 7.40E-61 | VFG013731 | gi:68536864 | Predicted | (fagC) putative iron ABC transport system, ATP-binding protein [ABC transporter (CVF516)] [Corynebacterium jeikeium K411] |
| CA2GL000189 | 41.2 | 1.30E-39 | VFG036556 | gi:385854783 | Predicted | (fbpC) iron(III) ABC transporter ATP-binding protein [ABC transporter (CVF197)] [Neisseria meningitidis M01-240355] |
| **CA2GL000255** | **42.9** | **6.40E-36** | **VFG036559** | **gi:313668880** | **Predicted** | **(fbpC) iron-uptake permease ATP-binding protein [ABC transporter (CVF197)] [Neisseria lactamica 020-06]** |
| CA2GL001485 | 45.1 | 4.20E-55 | VFG038840 | gi:507521851 | Predicted | (flmH) 3-oxoacyl-ACP reductase [Polar flagella (VF0473)] [Aeromonas hydrophila ML09-119] |
| CA2GL000374 | 47.4 | 2.40E-272 | VFG047710 | unknown | Predicted | (FN3523_0021) carbamoyl-phosphate synthase large chain [Pyrimidine biosynthesis (CVF845)] [Francisella cf. tularensis subsp. novicida 3523] |
| CA2GL000599 | 43.3 | 4.00E-99 | VFG047258 | unknown | Predicted | (FN3523_0439) N-acetylglucosamine-1-phosphate uridyltransferase/glucosamine-1-phosphate N-acetyltransferase [LPS (CVF834)] [Francisella cf. tularensis subsp. novicida 3523] |
| CA2GL000987 | 43.6 | 3.40E-48 | VFG046612 | unknown | Predicted | (FN3523_1292) Ribulose-phosphate 3-epimerase [Capsule (CVF833)] [Francisella cf. tularensis subsp. novicida 3523] |
| CA2GL000697 | 42.5 | 2.80E-73 | VFG047569 | unknown | Predicted | (FNFX1_0412) hypothetical protein [Purine (CVF839)] [Francisella cf. novicida Fx1] |
| CA2GL002026 | 100 | 7.60E-137 | VFG045678 | gi:397700125 | Predicted | (fsrA) FsrA response regulator [Fsr locus (CVF616)] [Enterococcus faecalis D32] |
| CA2GL002025 | 100 | 1.70E-136 | VFG045677 | gi:384513492 | Predicted | (fsrB) FsrB protein [Fsr locus (CVF616)] [Enterococcus faecalis OG1RF] |
| CA2GL002024 | 100 | 1.40E-195 | VFG002192 | gb\|NP_815517 | Verified | (fsrC) histidine kinase, putative [Fsr (VF0360)] [Enterococcus faecalis V583] |
| CA2GL002232 | 98.3 | 0.00E+00 | VFG043508 | gb\|NP_813892 | Verified | (fss1) Enterococcus faecalis surface protein Fss1, fibrinogen binding protein [Fibrinogen binding protein (AI271)] [Enterococcus faecalis V583] |
| CA2GL000434 | 91.9 | 0.00E+00 | VFG043509 | gb\|NP_816151 | Verified | (fss2) Enterococcus faecalis surface protein Fss2, fibrinogen binding protein [Fibrinogen binding protein (AI272)] [Enterococcus faecalis V583] |
| CA2GL002023 | 99 | 8.50E-292 | VFG045660 | gi:397700122 | Predicted | (gelE) gelatinase [Gelatinase (CVF612)] [Enterococcus faecalis D32] |
| CA2GL000925 | 42.8 | 4.50E-95 | VFG026433 | gi:383308019 | Predicted | (glnA1) glutamine synthetase [Glutamine synthesis (CVF311)] [Mycobacterium tuberculosis RGTB327] |
| CA2GL002537 | 72.1 | 4.70E-196 | VFG048851 | unknown | Predicted | (gnd) 6-phosphogluconate dehydrogenase [Capsule (CVF854)] [Klebsiella pneumoniae subsp. pneumoniae MGH 78578] |
| CA2GL000196 | 71.6 | 1.10E-212 | VFG012103 | gi:125975373 | Predicted | (groEL) chaperonin GroEL [GroEL (CVF403)] [Clostridium thermocellum ATCC 27405] |
| CA2GL000200 | 46.4 | 4.90E-29 | VFG032844 | gi:16801755 | Predicted | (gtcA) wall teichoic acid glycosylation protein GtcA [Cell wall teichoic acid glycosylation protein (CVF677)] [Listeria innocua Clip11262] |
| CA2GL002256 | 78.9 | 2.10E-133 | VFG005871 | gi:94991482 | Predicted | (hasC) UTP--glucose-1-phosphate uridylyltransferase [Capsule (CVF186)] [Streptococcus pyogenes MGAS10270] |
| CA2GL000817 | 40.1 | 2.20E-62 | VFG013192 | gi:170717476 | Predicted | (hitC) ABC transporter related [Haemophilus iron transport locus (CVF501)] [Haemophilus somnus 2336] |
| CA2GL002639 | 40.4 | 4.20E-37 | VFG013573 | gi:113460924 | Predicted | (hitC) iron(III) ABC transporter, ATP-binding protein [Haemophilus iron transport locus (CVF501)] [Haemophilus somnus 129PT] |
| CA2GL000403 | 46.3 | 1.30E-52 | VFG016229 | gi:30020330 | Predicted | (hlyIII) Hemolysin III [Hemolysin III (CVF560)] [Bacillus cereus ATCC 14579] |
| CA2GL000337 | 54.3 | 3.00E-112 | VFG005527 | gi:76788422 | Predicted | (htrA/degP) serine peptidase HtrA [Serine protease (CVF148)] [Streptococcus agalactiae A909] |
| CA2GL002698 | 41.3 | 2.60E-60 | VFG044190 | gi:49474051 | Predicted | (hutC) hemin ABC transporter, permease protein [direct heme uptake system (IA054)] [Bartonella quintana str. Toulouse] |
| CA2GL001889 | 42.2 | 2.90E-52 | VFG045346 | gb\|NP_933683 | Verified | (IlpA) immunogenic lipoprotein A [IlpA (VF0513)] [Vibrio vulnificus YJ016] |
| CA2GL002653 | 50.9 | 6.80E-105 | VFG037039 | gi:385850481 | Predicted | (katA) catalase [Catalase (CVF760)] [Neisseria meningitidis M04-240196] |
| CA2GL001179 | 54.6 | 1.20E-266 | VFG049190 | unknown | Predicted | (KOX_00005) protein disaggregation chaperone [T6SS-II (CVF861)] [Klebsiella oxytoca KCTC 1686] |
| **CA2GL001203** | **66.2** | **1.40E-150** | **VFG048790** | **unknown** | **Predicted** | **(KOX_25120) UDP-glucose 6-dehydrogenase [Capsule (CVF854)] [Klebsiella oxytoca KCTC 1686]** |
| **CA2GL000259** | **58.7** | **1.80E-73** | **VFG049038** | **unknown** | **Predicted** | **(KPN2242_15480) hypothetical protein [LPS rfb locus (CVF857)] [Klebsiella pneumoniae KCTC 2242]** |
| CA2GL002685 | 44.5 | 3.30E-107 | VFG006720 | gi:116873066 | Predicted | (lap) aldehyde-alcohol dehydrogenase protein [Listeria adhesion protein (CVF228)] [Listeria welshimeri serovar 6b str. SLCC5334] |
| CA2GL001749 | 70 | 0.00E+00 | VFG031960 | gi:347549024 | Predicted | (lap) putative alcohol-acetaldehyde dehydrogenase [Listeria adhesion protein (CVF228)] [Listeria ivanovii subsp. ivanovii PAM 55] |
| CA2GL002694 | 41.3 | 2.30E-68 | VFG031960 | gi:347549024 | Predicted | (lap) putative alcohol-acetaldehyde dehydrogenase [Listeria adhesion protein (CVF228)] [Listeria ivanovii subsp. ivanovii PAM 55] |
| CA2GL002258 | 50.7 | 8.40E-76 | VFG032463 | gi:525734762 | Predicted | (lgt) prolipoprotein diacylglyceryl transferase [Lipoprotein diacylglyceryl transferase (CVF248)] [Listeria monocytogenes J1-220] |
| CA2GL002539 | 41.3 | 9.10E-97 | VFG032638 | gi:386732117 | Predicted | (lisK) two-component sensor histidine kinase [LisR/LisK (CVF253)] [Listeria monocytogenes 07PF0776] |
| CA2GL002538 | 80.3 | 4.70E-101 | VFG006826 | gi:16803417 | Predicted | (lisR) two-component response regulator [LisR/LisK (CVF253)] [Listeria monocytogenes EGD-e] |
| CA2GL000595 | 41.5 | 4.60E-62 | VFG005232 | gi:125718778 | Predicted | (lmb) Zn-porter lipoprotein, putative [Laminin-binding protein (CVF114)] [Streptococcus sanguinis SK36] |
| CA2GL002194 | 41.8 | 2.40E-29 | VFG045566 | gb\|YP_096368 | Verified | (lpg2359) Dot/Icm type IV secretion system effector [Dot/Icm (VF0156)] [Legionella pneumophila subsp. pneumophila str. Philadelphia 1] |
| CA2GL002565 | 48.3 | 1.30E-86 | VFG006777 | gi:116872329 | Predicted | (lplA1) lipoyltransferase and lipoate-protein ligase family protein [Lipoate protein ligase A1 (CVF238)] [Listeria welshimeri serovar 6b str. SLCC5334] |
| CA2GL001292 | 63.3 | 3.50E-121 | VFG032200 | gi:347548334 | Predicted | (lplA1) putative lipoate protein ligase A [Lipoate protein ligase A1 (CVF238)] [Listeria ivanovii subsp. ivanovii PAM 55] |
| CA2GL000367 | 53 | 4.80E-32 | VFG032493 | gi:347549240 | Predicted | (lspA) putative signal peptidase II [Lipoprotein-specific signal peptidase II (CVF249)] [Listeria ivanovii subsp. ivanovii PAM 55] |
| CA2GL000780 | 45.2 | 5.10E-27 | VFG018246 | gi:59711152 | Predicted | (luxS) S-ribosylhomocysteinase [Autoinducer-2 (CVF628)] [Vibrio fischeri ES114] |
| CA2GL000059 | 41.9 | 2.00E-87 | VFG026392 | gi:507420368 | Predicted | (lysA) diaminopimelate decarboxylase [Lysine synthesis (CVF310)] [Mycobacterium abscessus subsp. bolletii 50594] |
| **CA2GL001772** | **42.2** | **4.10E-22** | **VFG019071** | **gi:169832565** | **Predicted** | **(lytA) lytic amidase (N-acetylmuramoyl-L-alanine amidase) [Choline-binding proteins (CVF122)] [Streptococcus pneumoniae Hungary19A-6]** |
| CA2GL000125 | 40.9 | 3.50E-27 | VFG016389 | gi:42784428 | Predicted | (lytR) membrane-bound transcriptional regulator LytR [Polysaccharide capsule (CVF567)] [Bacillus cereus ATCC 10987] |
| CA2GL002401 | 43.1 | 2.30E-60 | VFG016389 | gi:42784428 | Predicted | (lytR) membrane-bound transcriptional regulator LytR [Polysaccharide capsule (CVF567)] [Bacillus cereus ATCC 10987] |
| **CA2GL001190** | **42.2** | **4.00E-66** | **VFG016408** | **gi:49481246** | **Predicted** | **(lytR) membrane-bound transcriptional regulator LytR [Polysaccharide capsule (CVF567)] [Bacillus thuringiensis serovar konkukian str. 97-27]** |
| CA2GL000479 | 50 | 3.80E-88 | VFG016424 | gi:118480308 | Predicted | (manA) mannose-6-phosphate isomerase [Polysaccharide capsule (CVF567)] [Bacillus thuringiensis str. Al Hakam] |
| CA2GL001565 | 47.7 | 2.40E-233 | VFG018402 | gi:161505694 | Predicted | (mgtB) hypothetical protein [Mg2+ transport (CVF005)] [Salmonella enterica subsp. arizonae serovar 62:z4,z23:-- str. RSK2980] |
| CA2GL001100 | 48.8 | 7.60E-240 | VFG021055 | gi:207858995 | Predicted | (mgtB) Magnesium transport ATPase, P-type 2 [Mg2+ transport (CVF005)] [Salmonella enterica subsp. enterica serovar Enteritidis str. P125109] |
| CA2GL002399 | 40.2 | 2.50E-17 | VFG009424 | gi:119870217 | Predicted | (mgtC) MgtC/SapB transporter [Magnesium transport (CVF313)] [Mycobacterium sp. KMS] |
| CA2GL000106 | 46.6 | 1.10E-16 | VFG043551 | gi:15827894 | Predicted | (ML1683) histone-like protein [histone-like protein (Hlp)/laminin-binding protein (LBP) (AI354)] [Mycobacterium leprae TN] |
| **CA2GL002328** | **40.2** | **3.80E-21** | **VFG042130** | **gi:13475288** | **Predicted** | **(mlr6326) putative DNA invertase [T3SS (SS026)] [Mesorhizobium loti MAFF303099]** |
| CA2GL000930 | 45.7 | 4.40E-98 | VFG013515 | gi:148826007 | Predicted | (mrsA/glmM) predicted phosphomannomutase [Exopolysaccharide (CVF495)] [Haemophilus influenzae PittEE] |
| CA2GL001140 | 45.9 | 1.60E-27 | VFG031486 | gi:379746526 | Predicted | (ndk) nucleoside diphosphate kinase [Nucleoside diphosphate kinase (CVF660)] [Mycobacterium intracellulare ATCC 13950] |
| **CA2GL002339** | **41.1** | **1.00E-23** | **VFG004732** | **gi:87161840** | **Predicted** | **(nuc) thermonuclease precursor [Thermonuclease (CVF093)] [Staphylococcus aureus subsp. aureus USA300_FPR3757]** |
| **CA2GL001648** | **100** | **0.00E+00** | **VFG045650** | **gi:384512835** | **Predicted** | **(OG1RF_10871) cell wall surface anchor family protein [Ebp pili (CVF821)] [Enterococcus faecalis OG1RF]** |
| **CA2GL001647** | **100** | **1.90E-160** | **VFG045656** | **gi:384512836** | **Predicted** | **(OG1RF_10872) sortase [Ebp pili (CVF821)] [Enterococcus faecalis OG1RF]** |
| CA2GL001397 | 78.2 | 9.10E-185 | VFG046474 | unknown | Predicted | (OOM_0626) elongation factor [EF-Tu (CVF827)] [Francisella noatunensis subsp. orientalis str. Toba 04] |
| CA2GL002483 | 40.3 | 2.70E-126 | VFG006782 | gi:16804235 | Predicted | (oppA) hypothetical protein [Oligopeptide-binding protein (CVF240)] [Listeria monocytogenes EGD-e] |
| CA2GL001756 | 44.6 | 3.00E-141 | VFG032255 | gi:289435537 | Predicted | (oppA) oligopeptide ABC transporter substrate-binding protein [Oligopeptide-binding protein (CVF240)] [Listeria seeligeri serovar 1/2b str. SLCC3954] |
| CA2GL000602 | 41.8 | 7.20E-135 | VFG019366 | gi:217963642 | Predicted | (oppA) oligopeptide ABC transporter, oligopeptide-binding protein [Oligopeptide-binding protein (CVF240)] [Listeria monocytogenes HCC23] |
| CA2GL000067 | 42.8 | 1.90E-132 | VFG032247 | gi:386044509 | Predicted | (oppA) peptide/nickel transport system substrate-binding protein [Oligopeptide-binding protein (CVF240)] [Listeria monocytogenes 10403S] |
| CA2GL000349 | 42.9 | 1.40E-130 | VFG006785 | gi:116873629 | Predicted | (oppA) periplasmic oligopeptide-binding protein [Oligopeptide-binding protein (CVF240)] [Listeria welshimeri serovar 6b str. SLCC5334] |
| CA2GL001760 | 43 | 3.90E-30 | VFG016532 | gi:42561491 | Predicted | (oppF) oligopeptide ABC transporter, permease component [Capsule (CVF591)] [Mycoplasma mycoides subsp. mycoides SC str. PG1] |
| CA2GL001060 | 47.6 | 1.90E-40 | VFG013265 | gb\|NP_438428 | Verified | (orfM) deoxyribonucleotide triphosphate pyrophosphatase [LOS (CVF494)] [Haemophilus influenzae Rd KW20] |
| CA2GL001857 | 41.3 | 1.90E-08 | VFG040912 | gi:50122029 | Predicted | (outG) general secretion pathway protein G [out (SS212)] [Pectobacterium atrosepticum SCRI1043] |
| CA2GL000844 | 50.1 | 5.40E-154 | VFG005198 | gi:125717748 | Predicted | (pavA) Fibronectin-binding protein A, putative [Fibronectin-binding proteins (CVF113)] [Streptococcus sanguinis SK36] |
| CA2GL001102 | 45.6 | 8.60E-80 | VFG016502 | gi:47459418 | Predicted | (pdhB) pyruvate dehydrogenase E1 component beta subunit [PDH-B (CVF588)] [Mycoplasma mobile 163K] |
| CA2GL002716 | 41.7 | 2.00E-68 | VFG016502 | gi:47459418 | Predicted | (pdhB) pyruvate dehydrogenase E1 component beta subunit [PDH-B (CVF588)] [Mycoplasma mobile 163K] |
| CA2GL001063 | 48.5 | 1.70E-68 | VFG011639 | gi:118475479 | Predicted | (pebA) glutamine-binding protein [PEB1/CBF1 (CVF388)] [Campylobacter fetus subsp. fetus 82-40] |
| CA2GL001217 | 47.7 | 1.10E-74 | VFG005813 | gi:15901700 | Predicted | (piuA) iron-compound ABC transporter, iron-compound-binding protein [Pneumococcal iron uptake (CVF183)] [Streptococcus pneumoniae TIGR4] |
| CA2GL001834 | 80.5 | 1.10E-151 | VFG005356 | gi:116516442 | Predicted | (plr/gapA) glyceraldehyde-3-phosphate dehydrogenase, type I [Streptococcal plasmin receptor/GAPDH (CVF123)] [Streptococcus pneumoniae D39] |
| CA2GL000084 | 57.9 | 3.60E-105 | VFG019077 | gi:169832817 | Predicted | (plr/gapA) glyceraldehyde-3-phosphate dehydrogenase, type I [Streptococcal plasmin receptor/GAPDH (CVF123)] [Streptococcus pneumoniae Hungary19A-6] |
| CA2GL000791 | 48.7 | 9.60E-57 | VFG031738 | gi:169631126 | Predicted | (regX3) Sensory transduction protein RegX3 [RegX3 (CVF667)] [Mycobacterium abscessus ATCC 19977] |
| CA2GL000853 | 42.2 | 8.20E-40 | VFG031735 | gi:118619653 | Predicted | (regX3) two component sensory transduction protein RegX3 [RegX3 (CVF667)] [Mycobacterium ulcerans Agy99] |
| CA2GL000387 | 42.2 | 2.70E-51 | VFG031731 | gi:126433265 | Predicted | (regX3) two component transcriptional regulator [RegX3 (CVF667)] [Mycobacterium sp. JLS] |
| CA2GL001843 | 41.8 | 3.30E-172 | VFG009928 | gi:118471771 | Predicted | (relA) GTP pyrophosphokinase [(p)ppGpp synthesis and hydrolysis (CVF335)] [Mycobacterium smegmatis str. MC2 155] |
| CA2GL000896 | 80.2 | 3.80E-132 | VFG018664 | gi:157151373 | Predicted | (rfbA-1) glucose-1-phosphate thymidylyltransferase [Capsule (CVF186)] [Streptococcus gordonii str. Challis substr. CH1] |
| CA2GL001782 | 43.4 | 7.20E-35 | VFG045340 | gb\|NP_539653 | Verified | (ricA) Rab2 interacting conserved protein A [RicA (VF0414)] [Brucella melitensis bv. 1 str. 16M] |
| CA2GL000897 | 51.9 | 6.80E-45 | VFG007663 | gi:37678487 | Predicted | (rmlC) dTDP-6-deoxy-D-xylo-4-hexulose-3,5-epimerase [Capsular polysaccharide (CVF282)] [Vibrio vulnificus YJ016] |
| CA2GL000899 | 62.2 | 4.90E-98 | VFG006081 | gi:55823390 | Predicted | (rmlD) dTDP-4-keto-L-rhamnose reductase [Capsule (CVF186)] [Streptococcus thermophilus CNRZ1066] |
| CA2GL002048 | 99.4 | 5.20E-256 | VFG043442 | gi:29377518 | Predicted | (salA) secreted lipase, putative [Fibronectin-binding protein (AI159)] [Enterococcus faecalis V583] |
| CA2GL002733 | 98.2 | 1.40E-242 | VFG043443 | gi:29375030 | Predicted | (salB) secreted antigen, putative [Fibronectin-binding protein (AI160)] [Enterococcus faecalis V583] |
| CA2GL000095 | 45.8 | 1.10E-40 | VFG043456 | gi:22537734 | Predicted | (scpB) segregation and condensation protein B [Fibronectin-binding protein (AI186)] [Streptococcus agalactiae 2603V/R] |
| CA2GL000680 | 40.8 | 9.90E-120 | VFG009230 | gi:120404141 | Predicted | (secA2) SecA DEAD domain protein [Accessory secretion factor (CVF299)] [Mycobacterium vanbaalenii PYR-1] |
| CA2GL000892 | 51.9 | 1.70E-108 | VFG018674 | gi:157150246 | Predicted | (SGO_1723) RgpG [Capsule (CVF186)] [Streptococcus gordonii str. Challis substr. CH1] |
| CA2GL000080 | 56.8 | 3.10E-102 | VFG026984 | gi:499075370 | Predicted | (sigA/rpoV) RNA polymerase sigma factor SigA [Sigma A (CVF325)] [Mycobacterium avium subsp. paratuberculosis MAP4] |
| CA2GL001884 | 40.4 | 6.50E-55 | VFG034220 | gi:387606977 | Predicted | (sitC) iron ABC transporter permease [Iron/managanease transport (CVF459)] [Escherichia coli O44:H18 042] |
| CA2GL000090 | 50.4 | 3.40E-63 | VFG019080 | gi:182683690 | Predicted | (slrA) peptidyl-prolyl cis-trans isomerase, cyclophilin-type [Streptococcal lipoprotein rotamase A (CVF129)] [Streptococcus pneumoniae CGSP14] |
| CA2GL001802 | 49 | 1.20E-49 | VFG019080 | gi:182683690 | Predicted | (slrA) peptidyl-prolyl cis-trans isomerase, cyclophilin-type [Streptococcal lipoprotein rotamase A (CVF129)] [Streptococcus pneumoniae CGSP14] |
| CA2GL002403 | 50.3 | 5.20E-51 | VFG001867 | gb\|YP_096960 | Verified | (sodB) superoxide dismutase [SodB (VF0169)] [Legionella pneumophila subsp. pneumophila str. Philadelphia 1] |
| CA2GL002022 | 99.3 | 4.40E-168 | VFG045663 | gi:384513489 | Predicted | (sprE) SprE protein [SprE (CVF613)] [Enterococcus faecalis OG1RF] |
| CA2GL002052 | 48.3 | 7.70E-57 | VFG005379 | gi:24379545 | Predicted | (srtA) putative sortase [Sortase A (CVF130)] [Streptococcus mutans UA159] |
| CA2GL000898 | 81.1 | 1.40E-168 | VFG006022 | gi:116627988 | Predicted | (STER_1222) dTDP-D-glucose 4,6-dehydratase [Capsule (CVF186)] [Streptococcus thermophilus LMD-9] |
| CA2GL000900 | 61.4 | 4.40E-81 | VFG006079 | gi:116628179 | Predicted | (STER_1440) Glycosyltransferase involved in cell wall biogenesis [Capsule (CVF186)] [Streptococcus thermophilus LMD-9] |
| CA2GL000990 | 50 | 2.60E-60 | VFG032386 | gi:347549217 | Predicted | (stp) putative phosphoprotein phosphatase [Serine-threonine phosphatase (CVF245)] [Listeria ivanovii subsp. ivanovii PAM 55] |
| CA2GL002589 | 40.5 | 2.20E-43 | VFG030722 | gi:523912740 | Predicted | (sugC) ABC transporter, ATP-binding protein SugC [Trehalose-recycling ABC transporter (CVF651)] [Mycobacterium yongonense 05-1390] |
| CA2GL000178 | 47.4 | 2.40E-59 | VFG030678 | gi:145224436 | Predicted | (sugC) ABC transporter-like protein [Trehalose-recycling ABC transporter (CVF651)] [Mycobacterium gilvum PYR-GCK] |
| CA2GL001273 | 53.6 | 5.00E-100 | VFG030724 | gi:433649467 | Predicted | (sugC) carbohydrate ABC transporter ATP-binding protein, CUT1 family [Trehalose-recycling ABC transporter (CVF651)] [Mycobacterium smegmatis JS623] |
| CA2GL001794 | 40.2 | 6.30E-68 | VFG030724 | gi:433649467 | Predicted | (sugC) carbohydrate ABC transporter ATP-binding protein, CUT1 family [Trehalose-recycling ABC transporter (CVF651)] [Mycobacterium smegmatis JS623] |
| CA2GL001901 | 66.8 | 2.80E-158 | VFG018661 | gi:157150639 | Predicted | (tig/ropA) trigger factor [Trigger factor (CVF149)] [Streptococcus gordonii str. Challis substr. CH1] |
| CA2GL001218 | 49.4 | 8.60E-72 | VFG007257 | gi:28900515 | Predicted | (vctC) iron(III) ABC transporter, ATP-binding protein [Periplasmic binding protein-dependent ABC transport systems (CVF276)] [Vibrio parahaemolyticus RIMD 2210633] |
| CA2GL001220 | 42.7 | 2.70E-78 | VFG007245 | gi:15600997 | Predicted | (vctD) iron(III) ABC transporter, permease protein [Periplasmic binding protein-dependent ABC transport systems (CVF276)] [Vibrio cholerae O1 biovar El Tor str. N16961] |
| CA2GL001284 | 52.2 | 4.00E-68 | VFG006818 | gi:16803785 | Predicted | (virR) hypothetical protein [VirR/VirS (CVF252)] [Listeria monocytogenes EGD-e] |
| CA2GL000909 | 41.9 | 2.90E-36 | VFG047304 | unknown | Predicted | (wbtP) galactosyl transferase [LPS (CVF834)] [Francisella novicida U112] |
| **CA2GL001197** | **63.4** | **2.10E-71** | **VFG047304** | **unknown** | **Predicted** | **(wbtP) galactosyl transferase [LPS (CVF834)] [Francisella novicida U112]** |
| **CA2GL000904** | **44.7** | **4.50E-62** | **VFG026227** | **gi:126455338** | **Predicted** | **(wzt2) lipopolysaccharide ABC transporter ATP-binding protein [Capsule I (CVF645)] [Burkholderia pseudomallei 1106a]** |

The genes marked in bold blue font indicated that the genes did not overlap with strain CA1.
